# Assessment of the Presence and Strength of H-Bonds by Means of Corrected NMR

**DOI:** 10.3390/molecules21111426

**Published:** 2016-10-27

**Authors:** Steve Scheiner

**Affiliations:** Department of Chemistry and Biochemistry, Utah State University, Logan, UT 84322-0300, USA; steve.scheiner@usu.edu; Tel.: +1-435-797-7419

**Keywords:** β-sheet, CH··O H-bond, C5 dipeptide, aromatic amino acid, anti-electrostatic H-bond

## Abstract

The downfield shift of the NMR signal of the bridging proton in a H-bond (HB) is composed of two elements. The formation of the HB causes charge transfer and polarization that lead to a deshielding. A second factor is the mere presence of the proton-accepting group, whose electron density and response to an external magnetic field induce effects at the position of the bridging proton, exclusive of any H-bonding phenomenon. This second positional shielding must be subtracted from the full observed shift in order to assess the deshielding of the proton caused purely by HB formation. This concept is applied to a number of H-bonded systems, both intramolecular and intermolecular. When the positional shielding is removed, the remaining chemical shift is in much better coincidence with other measures of HB strength.

## 1. Introduction

The phenomenon of H-bonding is arguably the most well studied of all molecular interactions [1,2,3]. Following its initial conception and proposal of its earliest examples nearly a century ago, there have been countless papers that have appeared in the literature and a wealth of treatises, compendia, and books written on the subject. From the initial idea that involved a H atom bridging a pair of very electronegative atoms F, O, or N, the range of systems that contain such bonds has broadened quite a bit [4,5,6,7,8,9]. Much less electronegative proton donor atoms such as S, Cl, P, and C have been shown to participate in these bonds [10,11,12,13,14,15,16,17]. The electronegativity threshold for the proton-accepting atom has similarly dropped, and this electron source has expanded to include not only lone pairs, but also π and σ bonds [18,19].

Over the years, quite a number of markers have been developed [20,21,22,23,24,25] by which to identify the presence of a H-bond (HB). There is first a set of geometric criteria, in which the A and B atoms of the putative AH··B HB must lie within a cutoff distance. This threshold is an arbitrary one but is commonly taken to be the sum of the van der Waals radii of the A and B atoms. The θ(AH··B) angle tends toward linearity, but a certain tolerance of perhaps ±30° is permitted. Of course, these geometric criteria can only be evaluated if the structure is determined, so are typically limited to crystal or microwave structural evaluations. Spectroscopic measurements offer a second opportunity to examine a system for possible HBs. Within the context of IR and Raman spectra, the stretching frequency of the A-H covalent bond commonly undergoes a substantial red shift, and the band is intensified and broadened (this pattern can be reversed in certain HBs, largely those involving the CH donor [26,27,28,29,30]). The NMR signal of the bridging proton typically shifts downfield by several ppm upon formation of a HB [20,21,31]. These criteria offer quantitative information as well, in the sense that a shorter A··B distance signals a stronger HB [20], as do larger red shifts of the A-H stretching frequency or a greater NMR downfield shift [20].

The search for HBs took a major step forward as quantum chemical calculations became more accessible, particularly at high levels of theory. Computations of this sort offer a wide array of relevant tools. There is first the calculation of the interaction energy between the proton donor and acceptor molecules; a minimum of roughly 2 kcal/mol is a common threshold. This interaction can be dissected into various physical components. It is generally observed that the electrostatic attraction of HBs is somewhat greater than other attractive components such as induction or dispersion. Natural bond orbital (NBO) analysis [32,33] typically shows a transfer of charge from the electron donor lone pairs into the σ*(A-H) antibonding orbital, which accounts for the observed stretch and red shift of the A-H bond. Atoms-in-Molecules (AIM) treatment [34,35,36] presents a bond path from H to B, with an electron density and associated density Laplacian above a certain pair of thresholds. Like their experimental analogs, these computational measures also offer quantitative assessments of the strength of a given HB.

While the above means of identification of a HB, and estimate of its strength, have proven quite useful and even definitive over the years, their implementation within an intramolecular HB can be problematic. Many of the aforementioned measures refer to shifts from a non-bonded to a H-bonded situation. The non-bonded situation is simply defined in an intermolecular context, where the two molecules are assessed prior to their being allowed to interact with one another. However, what exactly should be taken as the non-bonded situation when both proton donor and acceptor reside on the same molecule? How can the internal HB be broken without causing other structural and electronic changes which will in turn affect other parameters? The A-H stretching frequency serves as a simple example. If one were to break the putative AH··B bond so as to measure the non-bonded AH frequency, the entire geometry of the molecule would likely be altered, so the AH frequency would then be measured in a very different situation. Even in the seemingly simple case of a short AH··B distance within a crystal, how can one distinguish whether this close contact is the result of an attractive HB from the situation where overall crystal structural considerations force these two units into close coincidence despite the absence of any real attraction?.

This intramolecular conundrum is not limited to experiment, but also plagues such computational tools as the interaction energy. The problem is again that of identifying a situation where the intramolecular HB has been broken, but with no other changes forced upon the molecule. There have been a number of prescriptions for estimating the interaction energy of an intramolecular HB [37,38,39,40,41,42,43,44,45], even if in only an approximate way, but none can be considered unambiguous, and some provide somewhat contrary results when compared with one another [46,47,48]. Thus, even if computational methods are capable of distinguishing the presence of an intramolecular HB, there is some confusion as to how to quantify its strength. Beyond computations, there is perhaps a more urgent need to establish a means by which experimental measurements can identify, and hopefully quantify, intramolecular HBs.

An attempt was made in this direction recently [49] in a study which considered the effects of HBs on IR and NMR spectral data. It was shown that the red shift of the A-H··B HB is not necessarily indicative of an attractive interaction. Even as the R(A··B) distance is compressed beyond its normal equilibrium geometry, into a region that is clearly repulsive, the ν(A-H) stretching frequency continues to shift further and further into the red, similar to earlier results [50]. Likewise, red shifts were observed even when the two molecules were rotated so as to void the normal alignment of a HB. The NMR chemical shielding of the bridging proton was shown to have greater potential in this regard [49], following earlier attempts [45] to utilize induced ring currents. However, before this quantity can be used to assess HB strength, it must first be cleansed of a spurious artifact which can obscure its relation to the H-bonding phenomenon itself. The present communication explores the viability of this approach in the context of a number of very diverse situations.

The first type of system contains what appears to be an intramolecular CH··O HB, which appears in conjunction with a stronger NH··O HB. There was some question [43,51,52] as to whether the former indeed represents a true HB, and if so as to its ability to influence the geometry of the full molecule. The second system addresses the limits of angular deformation of a HB. Calculations have shown [53,54,55,56,57,58,59,60] that the C5 conformation of a dipeptide, containing what shows some evidence of being a highly strained internal NH··O HB, represents a minimum, competitive in energy with the C7 structure with a much less distorted HB. The β-sheet secondary structure of proteins is normally thought to be stabilized by NH··O HBs between adjacent polypeptide strands. However, there are some clear indications [61,62,63,64] that this structure owes at least some of its stability to CH··O HBs that supplement the NH··O interactions. On another front, there have been numerous suggestions in the literature [65,66,67,68,69,70,71,72] that, although weak, numerous CH··π HBs involving aromatic rings as proton acceptors, represent an underestimated contributor to protein stability. And finally, the idea that a pair of ions of like charge can overcome their Coulombic repulsion in order to form a stabilizing HB is counterintuitive to many. However, recent calculations [73,74] have suggested just that: a minimum on the potential energy surface is fully consistent with the geometric criteria of a HB that holds the complex together in opposition to strong electrostatic repulsive forces attempting to pull it apart.

## 2. Computational Methods

The method is based [49] on the notion that when placed between two atoms that are potentially H-bonded to one another, the chemical shielding of the bridging proton derives from two separate phenomena. In the first place, there is what might be called a positional effect σ_p_. The proton-accepting group in and of itself, has a certain electron density surrounding it, which will respond to external magnetic fields, even in the absence of a true HB, and in fact without even a proton donor present. This positional effect is entirely independent of any HB that might form. If, on the other hand, a HB occurs, along with it comes a certain charge transfer between the donor and acceptor, as well as polarizations internal to each. This electronic adjustment process is a direct product of the HB itself, and is thus termed the HB shift ∆σ_HB_. It is this latter component which ought to best report on the possible presence of a HB and its strength.

In order to evaluate the latter, desirable, quantity, one first calculates the total chemical shielding of the proton within a given AH··B complex. From this is subtracted the shielding of the proton when the acceptor group is absent, which provides the total chemical shift of the proton ∆σ_T_. This shift represents the sum of the positional and HB components. In order to calculate the positional effect, the entire proton donor group is removed from the system. The shielding is then computed at the precise location of the missing proton (via the use of a “dummy atom” at this position). When this positional shielding is subtracted from the total shift, one is left with the desired HB component of the shift, as in Equation (1).

∆σ_HB_ = ∆σ_T_ − σ_p_ (1)

All calculations were carried out via the Gaussian-09 code [75]. The level of theory applied to each system was chosen so as to be consistent with prior calculations; it is indicated in each section. The AIMALL program [76] was used to evaluate the electron density at each of the bond critical points within the Atoms-in-Molecules context.

## 3. Results

### 3.1. Interamide HBs

**Figure 1:**
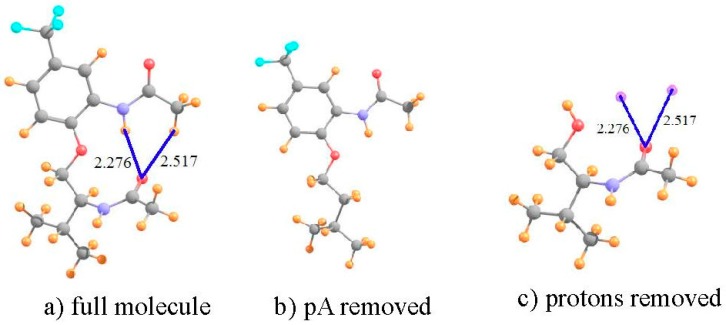
Structures of: (**a**) full molecule **I**; (**b**) **I** with lower amide group removed; and (**c**) **I** without upper phenyl ring and amide group. Small purple spheres in (**c**) indicate positions of upper amide protons involved in putative HBs. All distances in Å.

The first set of systems examined here is based upon prior experimental inquiries into the potential of H-bonding to influence the internal geometry of an organic system containing a pair of amide units [77]. Molecule **I**, represented in Figure 1a, almost assuredly contains a NH··O HB, but there was some question as to whether or not there is also a CH··O HB to the same carbonyl O proton-acceptor atom. Some recent calculations [22] addressed this issue, and found energetic and geometric data which were supportive of this notion. However, these calculations did not seek to provide the spectroscopic confirmation that might further buttress this case, and would assist experimentalists examining systems of this sort. It is just this sort of putative intramolecular HB that the method proposed earlier [49] is well suited to address.

To be consistent with the earlier calculations and optimized geometries [22], the calculations described below were similarly carried out at the B3LYP level, with the 6-31+G** basis set. The manner in which this analysis is carried out is illustrated for molecule **I**. This system shown in Figure 1a, has what appears to be a pair of intramolecular HBs. The NH··O HB is some 2.276 Å in length, and the putative CH··O HB is a bit longer, at 2.517 Å. Both utilize the carbonyl O atom of the lower amide unit as proton acceptor. Not only does the former HB take advantage of a stronger NH proton donor as compared to CH, and have a 0.24 Å shorter R(H··O), but the NH··O HB is closer to linearity, 165° vs. 152°. Thus, on several grounds, the NH··O HB ought to be stronger than the CH··O analog in this molecule.

Turning to the NMR data, the first row of Table 1 indicates the chemical shielding calculated for the two indicated protons in the full molecule. In order to obtain an estimate of what these shieldings would be in the absence of HBs, the entire amide unit proton acceptor was removed, and replaced by a H atom, as represented in Figure 1b. This proton was placed 1.095 Å from the C; the remainder of the geometry of the system was left unchanged. As indicated by the second row of Table 1, the shieldings are a bit larger without the proton acceptor group. The difference between them in the next row refers to the shift caused by adding the amide group. As may be seen, this shift is negative, toward smaller shielding, i.e., a deshielding caused by the formation of the intramolecular HBs. Note, however, that this shift is twice as large for the CH proton as for NH, running counter to the idea that the former is part of a considerably weaker HB.

However, what has not yet been considered is the shielding caused by the mere presence of the carbonyl O proton acceptor. In order to extract this positional shielding, the H-bonding protons were removed by deleting the entire upper portion of the molecule, illustrated in Figure 1c. A H atom was placed on the ether O atom (r_OH_ = 0.95 Å), after removing the phenyl ring (not expected to participate in any HB) plus the upper amide group containing the two H atoms of interest. As described in the earlier prescription, the shielding caused by the carbonyl O at the precise points where the two protons are located was assessed by replacing these two protons by dummy atoms, containing neither nucleus nor electrons nor orbitals. As indicated by the fourth row of Table 1, the electron density surrounding the peptide O atom would induce a deshielding of 0.166 ppm at the location occupied by the CH proton, but a shielding of 0.588 ppm at the NH position.

The shift that can be attributed to the density shifts caused by the formation of the intramolecular HB, exclusive of the mere presence of the proton acceptor, is computed by subtracting the simple influence of the O density (fourth row) from the full shift measured by comparing Figure 1a,b. The shift due to the HB itself is displayed in the fifth row of Table 1, and is −1.085 ppm for the CH proton versus −1.191 ppm for NH. Unlike the uncorrected shift, the larger deshielding of the latter is commensurate with the expectation that the NH··O HB ought to be the stronger of the two. Indeed, further confirmation of this distinction is provided by the values of the electron density at the AIM bond critical points, 12 mau for NH··O as compared to only 8 mau for the former.

It is clear then, that the consideration of the chemical shift in and of itself would provide a misleading conclusion that the CH··O HB is the stronger of the two, in direct contrast to chemical and geometric considerations. It is only after the removal from the shift of the influence of the mere presence of the peptide O, that the remaining shift, which is associated with the density perturbations caused by the HB formation itself, becomes consistent with other indicators.

**Figure 2:**
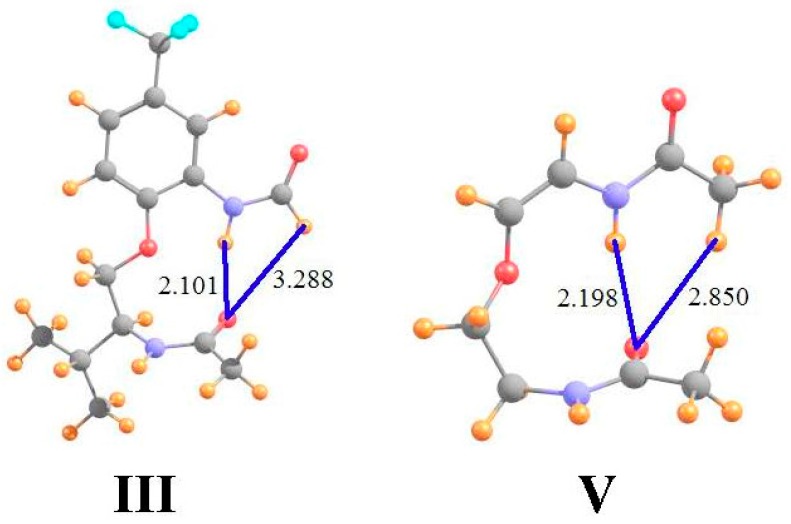
Geometries of molecules **III** and **V**, both derivatives of **I**. All distances in Å.

Data of a similar sort is provided in the ensuing columns of Table 1 for molecules **III** and **V** that are illustrated in Figure 2 (using the same molecular labeling as in the earlier work [22] for purposes of consistency). Molecule **III** is quite similar to **I**, except that the terminal methyl group on the upper amide has been replaced by a H atom. As is evident from the optimized geometry, this H is not in position to form a HB with the lower carbonyl O. Not only does it lie 3.3 Å from the O, but the θ(CH··O) angle is 105°, far from linearity. The chemical shieldings of the CH and NH protons are both about 22.8 ppm in **III**, as indicated in the first row of Table 1. However, when compared with the same quantities after the lower amide group has been removed, the HBs result in shifts of −0.21 and −1.12 ppm, respectively, suggesting the greater HB strength of the NH··O interaction. The fourth row of Table 1 shows that the presence of the peptide O, absent any interaction, would have only a small deshielding effect on the CH proton position, but would shield the NH proton by 0.64 ppm. When this effect is removed, the deshielding that can be attributed to the H-bonding phenomenon is an order of magnitude greater for NH··O than for CH··O. Indeed, AIM analysis suggests the absence of a CH··O HB, while the NH··O HB is quite strong, at 17 mau.

Molecule **V** in Figure 2 returns the methyl group, and the CH··O HB, while removing the extraneous parts of **I**, including the phenyl ring and the pendant isopropyl group. In comparison to **I**, the NH··O HB is shorter while CH··O is longer. Indeed, the AIM bond critical point densities in the last row of Table 1 confirm these effects upon HB strengths. The NMR chemical shifts also support the changes in HB strengths from **I** to **V**, with a smaller downfield shift for CH and a larger shift for NH. Note once again, however, how the correction to the shifts caused by removing the shielding of the peptide O have a significant effect, particular in enhancing the NH shift from −0.91 to −1.50 ppm. These corrected shifts are more in line with the AIM data, where in either case the NH/CH ratio is roughly 3.

### 3.2. Dipeptide Conformation

**Figure 3:**
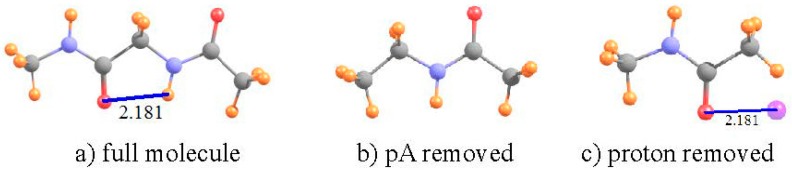
(**a**) Structure of C5 geometry of CH_3_NHCOCH_2_NHCOCH_3_ dipeptide. The left hand CH_3_NHCO group has been replaced by a methyl group in (**b**); and the right hand NHCOCH_3_ peptide replaced by H in (**c**). Purple sphere in (**c**) indicates position of H-bonding NH proton from (**a**). All distances in Å.

Over the years, numerous sets of calculations and experimental measurements [53,54,56,57,58,59,78,79,80] have shown that the two most stable conformations of a dipeptide can be characterized as C7 and C5, where 7 and 5 refer to the number of atoms that comprise the H-bonded ring. The C5 ring of diglycine, optimized at the B3LYP/6-31+G** level, is depicted in Figure 3 where the distance between the O and H atoms is 2.181 Å. While this distance is certainly short enough to sustain a HB, there is a good deal of angular distortion, with a θ(NH··O) angle of 107°, leading one to question the viability of such an intramolecular HB.

The data in Table 2 confirm that this HB does indeed exist, despite its deformation. There is a bond path between the H and O atoms, with a density at the critical point of 20 mau, quite substantial. With regard to NMR analysis, the shielding of the NH proton is 25.450 ppm in the full dipeptide in Figure 3. The HB was broken by removing the CONHCH_3_ unit on the left side of the molecule, replacing it with a methyl group, as indicated in Figure 3b. The comparison leads to a shift of −1.86 ppm associated with the HB. There is only a very small, −0.011 ppm, positional deshielding caused by the O atom at the location of the NH proton. Thus, the actual deshielding resulting from the HB process remains large, −1.752 ppm. In this case, then, the NMR data fully confirm the other indications that there is an intramolecular NH··O HB that helps stabilize the C5 geometry of the dipeptide, even with a high degree of angular distortion.

### 3.3. β-Sheet

One of the most prominent and widely occurring substructures within proteins [81] is the β-sheet, wherein the chain folds around so that one strand lies roughly parallel (or antiparallel) to the next strand. It is generally believed [82] that this type of structure is stabilized by NH··O HBs between one strand and its neighbor. However, there is also the distinct possibility that CH··O HBs are also present and add a substantial supplement to the interaction between the adjacent strands. This idea has been supported by a number of calculations over the years [62,63,64,83,84,85,86].

**Figure 4:**
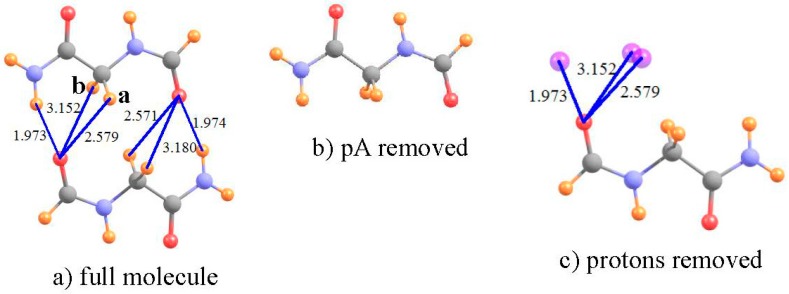
(**a**) Optimized NH_2_COCH_2_NHCHO dimer as model of segments of adjacent strands of antiparallel β-sheet. The lower dipeptide is removed in (**b**); and the upper deleted in (**c**). Purple spheres indicate locations of NH and CH protons from upper dipeptide. Distances are in Å.

In one such calculation [61], the relevant groups of the two strands were modeled by a pair of NH_2_COCH_2_NHCHO dipeptides, as shown in Figure 4a. Full MP2/6-31+G** geometry optimization of this pair led to a number of putative HBs, of both NH··O and CH··O type. Due to the near symmetry of the system, the following calculations focus on the three HBs on the left side of Figure 4a. The shielding of the NH and two CH protons are displayed in the first row of Table 3. In order to obtain the shifts in the absence of the proton acceptor, the entire lower dipeptide was removed, as in Figure 4b. The shifts induced by the lower dipeptide in Figure 4 are −2.801 and −0.869 for the NH and first of the two CH protons, respectively, with a negligible shift of the second CH. The lower dipeptide would in and of itself deshield the various protons by small amounts, as listed in the fourth row of Table 3. The NH and CH^a^ protons are thus deshielded by the HB itself, by −2.33 and −0.60 ppm, respectively; the second CH^b^ proton is not deshielded, and in fact experiences a small shielding. Consistent with these NMR data, the NH··O HB shows a large density at the bond critical point, the first CH^a^ half as much, and there is no bond path at all involving the second CH^b^. Both the NMR and AIM analyses are consistent with the idea that a CH··O HB is present between the two strands, although somewhat weaker than the NH··O interaction. In this instance, the removal of the positional shift is not critical, as it does not have a dramatic influence upon the conclusions, although it is responsible for numerical changes of as much as 0.5 ppm.

### 3.4. Aromatic Amino Acids

Recent years have witnessed a growing appreciation [65,66,67,68,69,70,71,72] of the presence of AH··π HBs to the π-systems of aromatic rings, and their influence upon protein structure. However, it is not always entirely clear that the location of an A-H group somewhere above the aromatic ring of a residue side chain truly signifies a stabilizing HB or if it is merely occupying a position forced upon it by the remainder of the protein structure, with little to no attraction between the two. It is thought that NMR spectra may be able to discriminate between these two possibilities. It is thus that attention is turned here to an analysis of what such NMR data might signify.

**Figure 5:**
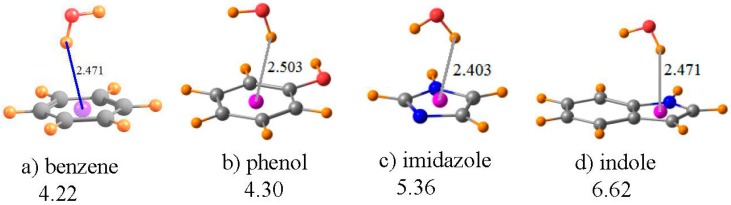
Optimized geometries of HOH situated above the planes of the indicated aromatic molecules. Purple sphere represents center of each ring. Numbers under the compound names refer to interaction energy in kcal/mol. Distances are in Å.

A water molecule was taken as a simple proton donor, which was placed above each of the aromatic rings of Figure 5, and the geometry optimized at the MP2/6-31+G** level, as in an earlier investigation of this issue [87]. The benzene ring in Figure 5a serves as the model of the aromatic ring of the Phe amino acid; phenol is the prototype of Tyr, imidazole as the functional group of His, and indole for Trp. The geometries in Figure 5 place the bridging proton between 2.4 and 2.5 Å above the center of each ring, as indicated. The chemical shift of the bridging proton of HOH is displayed in the first row of Table 4. The same quantity is reported in the next row for the water molecule, in its same geometry as in the complex, but with the aromatic system removed. The third row thus contains the shift in the NMR shielding of this proton caused by the presence of each aromatic system. Rather than the normal downfield shifts associated with HBs, these shifts are all positive, and substantially so, varying from a minimum of 1.5 ppm for imidazole up to a maximum of 2.5 ppm for benzene. Note also that the magnitude of this shift seems to have little regard for the interaction energy of each dimer, listed in the last row of Table 4. For example, the complex of benzene with water is the weakest, yet has the largest shift.

However, as in the earlier cases, this shift is a composite of: (i) the mere presence of the aromatic ring and the currents resulting from an external electric field; and (ii) the polarizations and charge transfers that accompany the formation of a HB. The fourth row of Table 4 contains item (i), i.e., the chemical shielding at the position of the bridging proton when the water molecule is removed from the system. Note that, in all cases, this is a large positive quantity, consistent with the shielding above an aromatic system due to the electric currents induced by a magnetic field. When this positional shielding is subtracted from the full shift, one derives the shift due to the HB itself. As indicated in the next row of Table 4, these quantities are all negative, consistent with normal expectations of a HB. Moreover, the amount of this deshielding corresponds reasonably well with the interaction energies, with the smallest quantities for benzene and the largest for indole.

It is worth stressing that these OH··π geometries represent a situation wherein it is absolutely necessary to remove the positional chemical shielding. The failure to correct this artifact yields proton chemical shifts of the wrong sign: upfield instead of the downfield shifts associated with true HBs. Needless to say, the magnitudes of these uncorrected shifts bear little resemblance to the actual strengths of the HBs.

### 3.5. Anti-Electrostatic HBs

While most HBs pair up two neutral molecules, there are also a range of systems where one or the other is electrically charged [88]. With either a cationic proton donor, or anionic acceptor, the interaction is usually very substantially enhanced. Even stronger are the so-called salt bridges between a pair of oppositely charged ions. However, the question has arisen of late as to whether a pair of molecules can engage in an attractive HB if both entities are of like charge [73,74,89,90,91,92,93]. One’s chemical intuition might suggest a negative answer, as the charges of the two subunits ought to strongly repel one another, preventing a close enough approach for a HB to form. On the other hand, if the two groups are somehow pushed close enough together, might the attractive forces of a forming HB overcome the overall Coulombic repulsions?

Recent work has suggested that just this latter scenario is possible, at least in certain instances [73,74]. As the charged proton donor and acceptor are pushed together, the potential is initially repulsive. However, once they approach to a threshold distance, the force becomes an attractive one. This latter attraction is insufficient to make the paired complex more stable than the fully separated pair of ions, but rather is capable of creating only a local minimum in the surface, even if only a shallow one. In other words, if one were to begin with the complex, as the two subunits are pulled apart, there is a small energy barrier which must be overcome, after which the potential is purely repulsive, taking the system to a fully separated pair, of lower energy than the complex, which may be considered as a metastable structure rather than as a global minimum.

**Figure 6:**
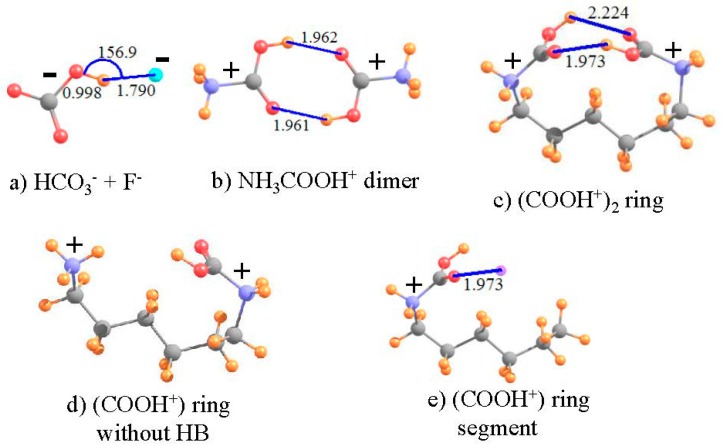
Pairs of like-charged ions; distances in Å, angles in degrees. Purple sphere in (**d**) indicates position of H-bonding proton from right hand COOH group.

What do various facets of the wave function and such observable properties as NMR spectra have to say about such a metastable complex pairing ions of like charge? In order to answer this question, two such “anti-electrostatic” HBs [73] were considered here, taken from recent examples in the literature. The HCO_3_^−^ anion is both negatively charged and contains a OH group which can potentially serve as proton donor, despite the overall negative charge. F^−^ is a very highly compact negatively charged species, which ought to resist the approach of any other anion. As in the earlier calculations [73], the structure was fully optimized at the MP2 level with the aug-cc-pVTZ basis set, and led to the geometry pictured in Figure 6a, which has the geometrical characteristics of a HB. The R(OH··F) distance is a rather short 1.790 Å, and the bond is close to linear, with θ(OH··F) = 157°. In addition, in common with most HBs, the covalent r(OH) bond is stretched by 0.033 Å, relative to the optimized HCO_3_^−^ monomer. Nonetheless, this bonded pair lies 55.6 kcal/mol higher in energy than does the fully separated pair, verifying the idea of a metastable complex. As reported in Table 5, the isotropic shielding of the bridging proton shifts downfield by 6.025 ppm in the complex, further strong evidence of the presence of a HB. The shielding at the position of this proton, caused solely by the presence of the fluoride is +0.862 ppm. Thus, the deshielding of this proton in the complex is even larger, −6.887 ppm, when evaluated as the deshielding caused by density perturbations caused by the HB, exclusive of the mere presence of the proton acceptor F^−^. As a final estimation of the HB strength, the electron density at the H··F critical point is 32.7 mau, quite a large quantity, and indicative of a true HB.

The same sort of analysis can be applied to a pair of cations. The potential energy surfaces of a pair of carboxylic acids, each containing a cationic NH_3_^+^ tail, were shown [74] to contain a metastable minimum, whose geometry contained what appeared as a pair of OH··O HBs. The authors were able to show that the full potential could reasonably accurately be described as the sum of: (i) a purely Coulombic repulsion between a pair of generic positive charges; and (ii) a typical HB potential. In order to more fully examine the possible presence of a HB in an anti-electrostatic complex of this type, the dimer pictured in Figure 6b, with each subunit containing both the cationic tail and -COOH group was examined here, applying the M06-2X functional with a aug-cc-pVTZ basis set [74]. As is clear from the geometry, there appears to indeed be a pair of equivalent OH··O HBs holding together this dimer, with R(H··O) distances of 1.962 Å. On the other hand, this structure is not a global minimum, but rather a metastable local minimum, 40.8 kcal/mol less stable than the pair of separated cations. As indicated by the second column of Table 5, the shielding of the two bridging protons in the complex is 20.948 ppm, some 2.165 ppm smaller than that of each NH_3_COOH^+^ monomer, in isolation from its partner. Each monomer would induce a positional deshielding at the location of the partner molecule’s proton of −0.129 ppm, so the deshielding caused by the HB density shifts amounts to −2.036 ppm, confirming the presence of a true HB. This contention is confirmed by the AIM critical point density of 20.9 mau in the last row of Table 5.

Both of the above systems contain an intermolecular HB. One can construct an analogous system with an intramolecular HB by connecting the two N atoms of Figure 6b by an aliphatic chain. Such a chain of 6 CH_2_ units leads to the structure depicted in Figure 6c. Note that the entire ring system is doubly charged, with each positive charge centered formally on the N atoms, parallel to the intermolecular analog in Figure 6b. The size of the ring was guided by the goal of engaging an interaction between the two COOH units as in the dimer. With only three CH_2_ units, the two -COOH units were unable to come close enough together due to steric strain within the ring. A larger ring, containing eight CH_2_ units placed the -COOH groups far removed from one another, with no internal interaction. This attraction is present with 6 such CH_2_ units, where it may be seen that there is a bit of distortion from the perfectly symmetrical dimer in Figure 6b. One of the two OH··O HBs is much shorter than the other, 1.973 Å vs. 2.224 Å. Due also to its lesser distortion, focus is placed on the shorter, and presumably stronger, of the two HBs in Figure 6c.

The NMR data for this intramolecular HB are listed in the final column of Table 5. As a reference point to the same system without any such HB, the lefthand -COOH unit of Figure 6c was replaced by a H atom (r_NH_ = 1.03 Å), as shown in Figure 6d. The shielding and shift of the proton in the intramolecular ring system are not entirely different than those in the preceding column, for the corresponding intermolecular cation-cation interaction. The chemical shift of the bridging proton is −2.391 ppm, compared to −2.165 ppm for the intermolecular HB. In order to generate a system wherein one can assess the positional shielding provided by the presence of the proton-acceptor unit at the location of the bridging proton, the -NH_2_COOH^+^ segment on the right side of the ring was removed, leaving behind HOOC-NH_2_-(CH_2_)_5_CH_3_^+^ in precisely the same geometry as the full ring containing the pair of -COOH groups, as displayed in Figure 6e (with r_CH_ = 1.095 Å). The shielding produced by the left-hand carboxyl group amounts to +0.101 ppm, opposite in sign to the intermolecular situation, but still small in magnitude. Upon removal of this quantity, the shift caused by the HB phenomenon itself is calculated to be −2.491 ppm. The magnitude of this term, as well as the large density at bond critical points (ρ_BCP_) of 21.0 mau, confirms the presence of a fairly strong HB in the intramolecular system, not very different than that calculated in the intermolecular HB pair. It is gratifying to see this strong parallel between the intramolecular HB and the intermolecular analog which is much more amenable to evaluation of the interaction energy.

It would be tempting to try to correlate the corrected NMR chemical shifts with bond critical point densities. However, since the systems examined here are so diverse, encompassing all sorts of HBs, neutral and charged, CH··O and NH··O, and with both lone pair and π-systems as proton acceptors, any such correlation would necessarily be a weak one. Indeed, an attempted correlation across this entire spectrum of HBs leads to a correlation coefficient of only 0.67. What is most important, however, is that within any subset of similar systems, this correlation is considerably stronger, and the two sorts of data provide parallel information.

## 4. Summary and Discussion

The earlier analysis [22] of the possibility of an intramolecular CH··O HB in molecule **I** and some of its derivatives had suggested its presence by a number of geometric criteria. Some derived energetic criteria, as well as analysis of the wave function, e.g., NBO, were also applied. However, most of these sorts of properties are not accessible to experimental measurement, so must rely purely on quantum chemical calculations. NMR chemical shifts, on the other hand, represent a workhorse of experimentalists in general, and in particular within the field of HBs. The calculated NMR shielding shows that in the case of these molecules, the downfield shifts of the CH and NH protons confirm their participation in true HBs. On the other hand, a simple measurement of the shifts can lead to misleading interpretation as to the relative strengths of the NH··O and CH··O HBs. In molecule **I**, for example, the CH proton is shifted downfield by twice as much as the NH proton. However, this measurement does not take into account the positional shift that is due purely to the presence of the carbonyl O acceptor atom, prior to, and exclusive of, any HB formation. The electron density around this O atom, and the currents that arise in response to an external magnetic field, deshield any atom placed in the CH position, while a shielding occurs at the NH proton position. The subsequent formation of the HB, and its deshielding effect upon a bridging proton, is therefore aided by the former deshielding, but in contrast, must fight against the latter shielding. The actual effects of the charge transfer and polarization caused by HB formation, after removal of the simple positional shielding, are much more in line with the expectation of a stronger NH··O vs. CH··O, as well as other numerical measures of bond strength such as AIM analysis. Indeed, this phenomenon is not limited to **I**, but occurs in several of its derivatives as well. The computation and removal of the simple positional shielding thus yields a far more quantitative measure of intramolecular HB strength.

These positional effects are quite small in the C5 conformation of the model dipeptide, so its removal is not critical. In either case, there is strong evidence of a true HB within this structure, which is in large part responsible for the fact that the C5 dipeptide represents a minimum on the full potential energy surface, even if not necessarily the global minimum. With regard to the interactions between adjacent strands of the model β-sheet of proteins, the chemical shifts induced in the NH and CH protons of one strand by the presence of the other suggest the existence of one NH··O and one CH··O HB. The presence of the proton acceptor strand, exclusive of any charge transfer or polarization, induces a deshielding of any atom placed in the NH or CH proton positions, larger for NH than for CH. Since the full deshielding is fairly large, the removal of this positional deshielding is not critical to the finding that both represent true HBs, but does provide a more quantitatively accurate measure of their binding strength, by as much as 30%.

The magnetic currents within an aromatic system are well known to effectively shield any atom placed directly above the ring. Such shielding can obscure the deshielding of a bridging proton normally associated with the formation of a HB. Indeed, in the systems studied here of models of four aromatic protein side chain residues, the aromatic shielding above the ring is quite large, leading to an upfield shift of a OH··π H-bonding proton for each and every such ring. This shift has no systematic correlation with the strength of the H-bonding interaction. However, when the ring current shielding is calculated and subtracted, the remaining shift is in the correct downfield direction. Moreover, this corrected deshielding correlates quite nicely with the strength of the HB.

There is lingering controversy concerning the question as to whether a shallow minimum on the potential energy surface of systems comprising a pair of like-charged ions represents a true HB or is an indication of some other phenomenon. Calculation of the NMR chemical shifts of the protons in these systems leads to quite negative values, consistent with the idea of a HB. In the case of a pair of anions, the positional shift in HCO_3_^−^··F^−^ is positive. However, the amount of the shift caused by the HB phenomenon itself is negative and much larger in magnitude, so the measured shift would be quite negative, in this case −6 ppm. The positional shifts in the systems containing a pair of cations, whether intermolecular or intramolecular, are rather small, so the full computed shift is a good estimate of the change caused by the HB itself. In all cases of these so-called anti-electrostatic HBs, there is every indication of a true HB holding the system together, in spite of a sizable Coulombic repulsion.

Of course, calculation of NMR chemical shifts is not the only means of assessing either the presence or strength of a HB. However, this spectroscopic technique is widely available to experimentalists, and has been used quite commonly for this purpose. The work described above offers qualified encouragement to these efforts when applied in an intramolecular setting. It is important first to establish a reference point when calculating the chemical shift. A system must be chosen in which there is no possibility of a HB, yet one which remains as faithful as possible to the original system of interest.

The second point is that one must recognize that the measured shift of the proton is due not only to the charge transfer and polarization of the electron density associated with the H-bonding phenomenon, but also to the simple presence of the proton acceptor group, even in the absence of any such attractive interaction. In some cases, the latter positional effect is a small one, and any downfield shift of the proton’s signal can be taken as a strong affirmation of a true HB. However, there are other cases which are not so simple. In the first place, this positional effect can be either positive or negative, i.e., shielding or deshielding. The sign of this quantity is not a simple function of the nature of the proton acceptor group. Rather, even the sign can change depending upon the precise location of the site of interest. The interamide HBs in Table 1, for example, showed a positional deshielding at the site of the CH proton, and shielding at the NH proton position, both interacting with the same exact O atom.

The most dramatic such situation, as described here, refers to a OH··π HB, wherein the bridging proton is located above an aromatic ring. The large shielding effect of the ring currents overwhelms the smaller deshielding of the HB process. Protons engaged in such a OH··π HB therefore shift upfield, in direct contrast to the usual downfield shift of H-bonding protons. It is only when the former positional shielding is evaluated and subtracted from the total that the actual HB phenomenon becomes visible, as the anticipated downfield shift. More than that, this corrected deshielding offers a quantitative measure of the HB strength, something that the uncorrected quantity cannot do.

It is thus hoped that future attempts to identify the presence of HBs via NMR spectroscopy will take these considerations into account. There is frequently more going on than is visible to the naked eye. It would not be difficult to assess the magnitude and sign of the positional shielding by a straightforward quantum chemical calculation of the sort described here. This evaluation would allow a more definitive statement to be made about the presence of such a bond, and indeed even concerning its strength. This sort of consideration is true not only for intermolecular HBs, which are the most common ones examined, but for intramolecular interactions as well.

## Figures and Tables

**Table 1:** NMR isotropic shielding (ppm) of molecules and indicated derivatives shown in Figure 1 and Figure 2, along with density at bond critical points (ρ_BCP_), in 10^−3^ au (mau).

	I ^a^		III ^b^		V ^b^	
	CH	NH	CH	NH	CH	NH
full molecule	28.912	23.078	22.882	22.809	29.592	23.438
pA removed	30.163	23.682	23.091	23.930	30.349	24.350
shift	−1.251	−0.603	−0.209	−1.121	−0.756	−0.912
protons removed	−0.166	+0.588	−0.048	+0.640	−0.101	+0.588
shift due to HB	−1.085	−1.191	−0.161	−1.761	−0.656	−1.500
ρ_BCP_	8	12	-	17	5	14

^a^ illustrated in Figure 1; ^b^ illustrated in Figure 2.

**Table 2:** NMR isotropic shielding (ppm) of C5 dipeptide conformation shown in Figure 3, along with density at bond critical point (ρ_BCP_), in 10^−3^ au.

	C5
full molecule	25.450
pA removed	27.312
shift	−1.862
proton removed	−0.011
shift due to HB	−1.752
ρ_BCP_	20.0

**Table 3:** NMR isotropic shielding (ppm) of models of the β-sheet structure in proteins shown in Figure 4, along with density at bond critical points (ρ_BCP_) in 10^−3^ au.

	NH	CH^a^	CH^b^
full molecule	23.947	27.297	28.071
pA removed	26.748	28.166	28.067
shift	−2.801	−0.869	+0.004
protons removed	−0.468	−0.269	−0.066
shift due to HB	−2.333	−0.600	+0.070
ρ_BCP_	20.6	9.1	-

**Table 4:** NMR isotropic shielding (ppm) of OH··π complexes shown in Figure 5, along with density at bond critical points (ρ_BCP_) in 10^−3^ au, and interaction energy (−∆E).

	Benzene	Phenol	Imidazole	Indole
full dimer	33.071	32.688	32.308	33.055
pA removed	30.546	30.743	30.788	30.741
shift	2.524	1.945	1.520	2.314
HOH removed	3.189	2.818	2.600	3.448
shift due to HB	−0.665	−0.873	−1.081	−1.134
ρ_BCP_	8.2	8.4	9.5	8.8
−∆E (kcal/mol)	4.22	4.30	5.36	6.62

**Table 5:** NMR isotropic shielding (ppm) of ion-ion structures shown in Figure 6, along with density at bond critical points (ρ_BCP_) in 10^−3^ au.

	HCO_3_^−^ + F^−^	(NH_3_COOH^+^)_2_	(COOH^+^)_2_ Ring ^a^
full dimer	21.101	20.948	21.645
pA removed	27.125	23.113	24.036
shift	−6.025	−2.165	−2.391
pD removed	+0.862	−0.129	+0.101
shift due to HB	−6.887	−2.036	−2.491
ρ_BCP_	32.7	20.9	21.0

^a^ see Figure 6c–e.
